# Prospective study of the behavior of totally implantable catheters in female oncology patients with mammary hypertrophy

**DOI:** 10.31744/einstein_journal/2026AO1922

**Published:** 2026-03-27

**Authors:** Maria Fernanda Cassino Portugal, Bruno Jeronimo Ponte, Carolina Carvalho Jansen Sorbello, Thulio Fernandes de Souza, Amanda Indig Pinheiro, Andressa Cristina Sposato Louzada, Dafne Braga Diamante Leiderman, Cynthia de Almeida Mendes, Nelson Wolosker

**Affiliations:** 1 Hospital Israelita Albert Einstein São Paulo SP Brazil Hospital Israelita Albert Einstein, São Paulo, SP, Brazil.; 2 Hospital Israelita Albert Einstein Faculdade Israelita de Ciências da Saúde Albert Einstein São Paulo SP Brazil Faculdade Israelita de Ciências da Saúde Albert Einstein, Hospital Israelita Albert Einstein, São Paulo, SP, Brazil.

**Keywords:** Catheterization, central venous, Vascular access devices, Mammaplasty, Neoplasms, Breast, Hypertrophy

## Abstract

**Objective:**

This study aimed to evaluate the change in the position of totally implantable venous access devices in patients with mammary hypertrophy compared to patients without this condition, in both supine and upright positions.

**Methods:**

This was a prospective study involving 116 female patients who underwent implantation of totally implantable venous access devices in the internal jugular vein with reservoir placement in the infraclavicular region of the chest wall. Patients with mammary hypertrophy were compared to a Control Group of patients with normotrophic breasts. Mammary hypertrophy was defined on the basis of the Sacchini index. The catheter tip was placed at the cavoatrial junction. The distances between the tip of the catheter and carina were compared intraoperatively and postoperatively. Measurements were performed using a radiopaque ruler intraoperatively under fluoroscopy and postoperatively with posteroanterior chest radiography in the upright position.

**Results:**

Of the 116 female patients, 53 (46.7%) had mammary hypertrophy, and 63 constituted the Control Group (53.3%). Displacement of the catheter tip in the cranial direction correlated with the previous presence of a peripherally inserted central catheter on the same side as the totally implantable venous access device (p=0.042). In contrast, caudal displacement correlated with non-deforming surgery on the chest wall (p=0.002). Patients with mammary hypertrophy did not show a higher incidence of catheter tip displacement than those with normotrophic breasts (p=0.182).

**Conclusion:**

Mammary hypertrophy, assessed using the Sacchini index, did not significantly correlate with catheter tip displacement.

## Highlights

Mammary hypertrophy was not associated with postoperative totally implantable venous access device catheter tip displacement.

Upright and supine chest radiography with a radiopaque ruler allowed precise quantification of catheter tip displacement.

Non-deforming chest wall surgery and a history of peripherally inserted central catheters history were significantly associated with catheter tip migration.

## In Brief

This prospective study evaluated whether mammary hypertrophy influences the postoperative displacement of totally implantable venous access device catheter tips. By comparing patients with and without mammary hypertrophy in both the upright and supine positions, the analysis demonstrated no significant association between mammary hypertrophy and catheter tip migration.

## Graphical abstract

**Figure:**
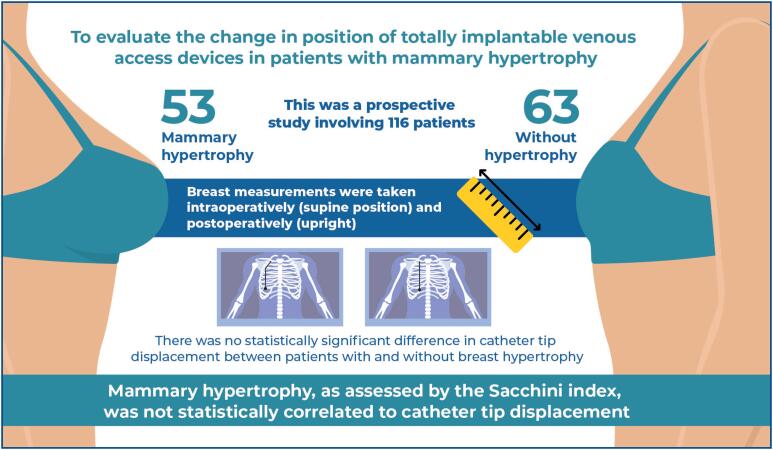

## INTRODUCTION

Long-term totally implantable venous access devices (TIVADs) are widely used in clinical practice, particularly in patients requiring frequent venous access such as those receiving chemotherapeutic agents that are either vesicants or unsuitable for peripheral administration. These devices are most commonly used for patients with cancer undergoing chemotherapy to preserve the superficial venous system and improve their quality of life.^(1-4)^

The proper functioning of TIVADs depends on accurate catheter positioning and the absence of deformation during use.^(5)^ To better understand the catheter positioning dynamics, Vesely et al. investigated the behavior of centrally inserted catheters and identified two key anatomical factors that influence tip migration in large-bore catheters placed via the subclavian or internal jugular veins.^(6)^ First, transitioning from a supine to an upright position results in the elongation of the central veins due to abdominal content displacement, which affects the position of the catheter tip relative to the right atrium.^(6)^ Second, the caudal displacement of the thoracic wall in an upright posture causes a cranial shift of the catheter tip, pulling it away from the cavoatrial junction.^(6)^ Studies assessing catheter movement under these conditions have reported changes of up to 3.2 cm,^(7)^ with greater effects observed in women and obese patients, particularly for larger catheters.^(8)^

Mammary hypertrophy is a common condition in women characterized by an increase in glandular tissue during puberty or excessive adipose tissue deposition.^(9)^ This results in increased breast weight and consequent caudal traction on the chest wall.^(6)^ Based on this observation, it is often hypothesized that patients with mammary hypertrophy may be at a higher risk of catheter tip migration following TIVAD implantation due to enhanced mechanical influence on the thoracic wall when in an upright position.

## OBJECTIVE

The objective of this study was to evaluate positional changes in totally implantable venous access devices catheter tips in both supine and upright positions in patients with mammary hypertrophy compared to those without the condition. This study also aimed to determine whether mammary hypertrophy was associated with a greater degree of catheter tip displacement.

## METHODS

### Study design and patient enrolment

This prospective study included consecutive female patients who underwent TIVAD implantation at a cancer hospital between August 2020 and August 2022.

This study adhered to the principles outlined in the Declaration of Helsinki and was approved by the Ethics Committee of *Hospital Israelita Albert Einstein* (CAAE: 45305121.5.0000.0071; #6.176.001). Before participation, all patients received comprehensive information about the study objectives and procedures, and informed consent was obtained from each participant. All data were handled confidentially and were de-identified.

Patients were included if they were over 18 years of age, female, had a formal indication for the implantation, and consented to participate by signing an Informed Consent Form. The sample included patients who underwent the implantation in the internal jugular vein with reservoir placement in the infraclavicular region of the thoracic wall.

Patients who could not tolerate chest radiography in an upright position, had significant hematomas, or had reservoirs placed in locations other than the anterior thoracic wall were excluded.

### Data collection and subgroup analysis

The patients were stratified into two groups based on breast volume: those with mammary hypertrophy and a Control Group. Classification was performed using the Sacchini index, which was measured preoperatively in the operating room with the patient in an upright position.

The Sacchini index is an objective tape-based anthropometric measure designed to reduce subjectivity in the assessment of mammary hypertrophy.^(10)^ Two linear distances were measured: from the lateral border of the sternum to the nipple and from the inframammary fold to the nipple. The lateral border of the sternum was considered the midpoint between the xiphoid process and the manubrium.

The index was defined as the average of these two values. Breast values with an index between 9 and 11cm were classified as average, whereas those with measurements exceeding 11 cm were considered hypertrophic. Each breast was assessed independently, and patient allocation to the hypertrophy or Control Group was based on the Sacchini index measurements corresponding to the side of the planned device implantation.^(11)^

The sample size was calculated based on the results of a study by Kowalski et al.^(7)^ who reported a mean catheter tip displacement of 3.2cm (standard deviation (SD)=1.8cm) in the general population. Assuming that women with mammary hypertrophy exhibit a catheter tip displacement at least 1 cm greater than the carina, and considering a power of 80% and 95% confidence, a minimum of 51 women per group was determined to be necessary for the study.

Demographic and clinical data were collected for all included patients, including age (years), height (cm), weight (kg), body mass index (BMI), type of malignancy, history of previous breast or thoracic wall procedures, presence of a peripherally inserted central catheter (PICC) at the time of TIVAD implantation, history of prior catheter placement at the same site, history of deep vein thrombosis (DVT) at the intended implantation site, and puncture site.^(10,11)^

### Procedure technique

According to the institutional protocol, all TIVADs were implanted in the operating room with the patient in a supine position under sedation and local anesthesia. Venous access was consistently guided by ultrasound and catheter placement was performed under fluoroscopic guidance. Reservoirs were implanted in the infraclavicular region to maintain uniformity during the procedure.

The same device was used in all cases: Life-Port® 8Fr (IBEG, INC. Pinhais; Paraná, Brazil). Local anesthesia was induced using 2% lidocaine without epinephrine.

The choice of venous access site was determined intraoperatively using ultrasonography based on the following predefined criteria: vessel diameter, compressibility, and phasicity. The preferred sequences for access site selection were as follows: the right internal jugular vein, left internal jugular vein, right subclavian vein, and left subclavian vein. The tip of the catheter was preferably positioned in the cavoatrial region in all patients based on the height of the carina during fluoroscopy.

No complications during the procedure were documented.

## INTRAOPERATIVE CATHETER TIP POSITION DOCUMENTATION

**Figure 1:**
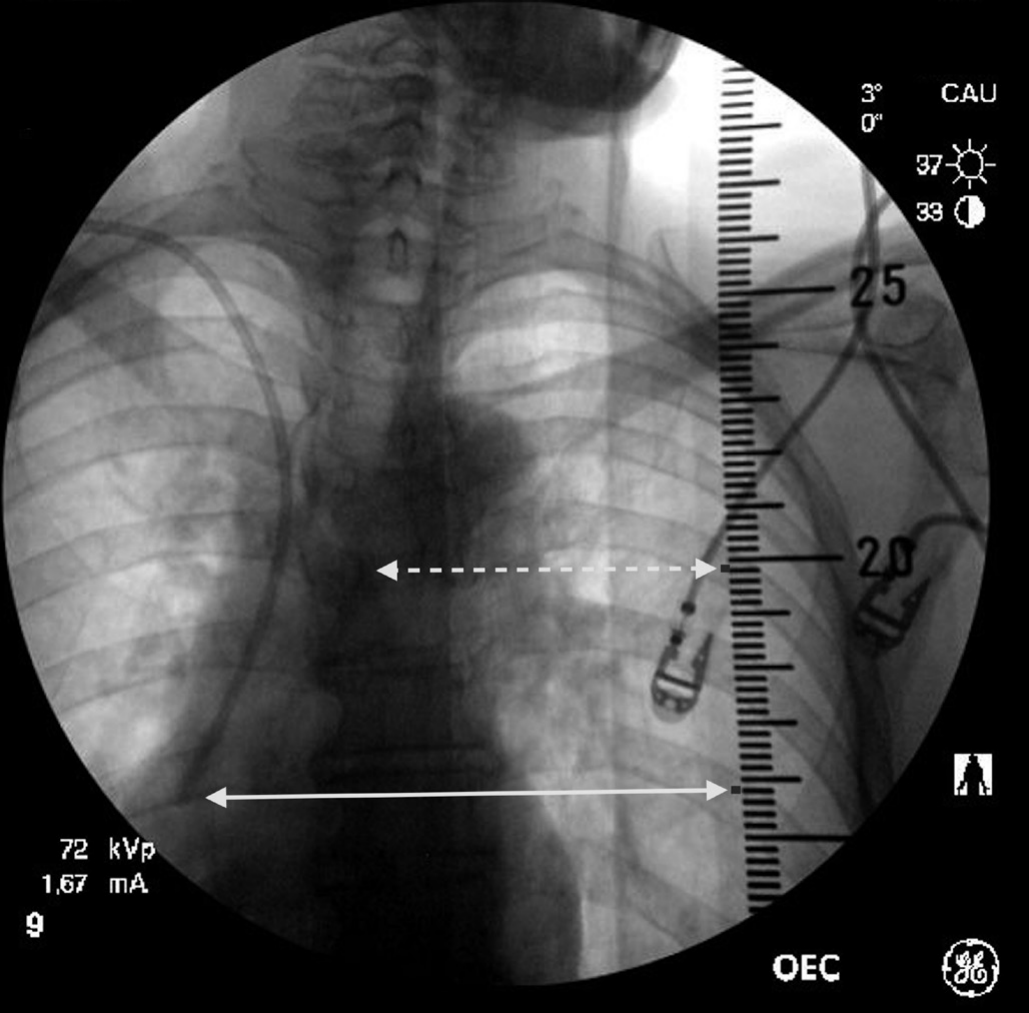
Intraoperative image of the measurement of catheter tip distance from the carina

The immediate postoperative position of the catheter tip was documented by using a fluoroscopic image obtained at the end of the procedure. A radiopaque ruler was positioned on the surgical table to measure the distance from the catheter tip to the carina in cm (Figure 1).

Plain arrow: catheter tip; Dashed arrow: carina.

### Postoperative upright catheter tip evaluation

**Figure 2:**
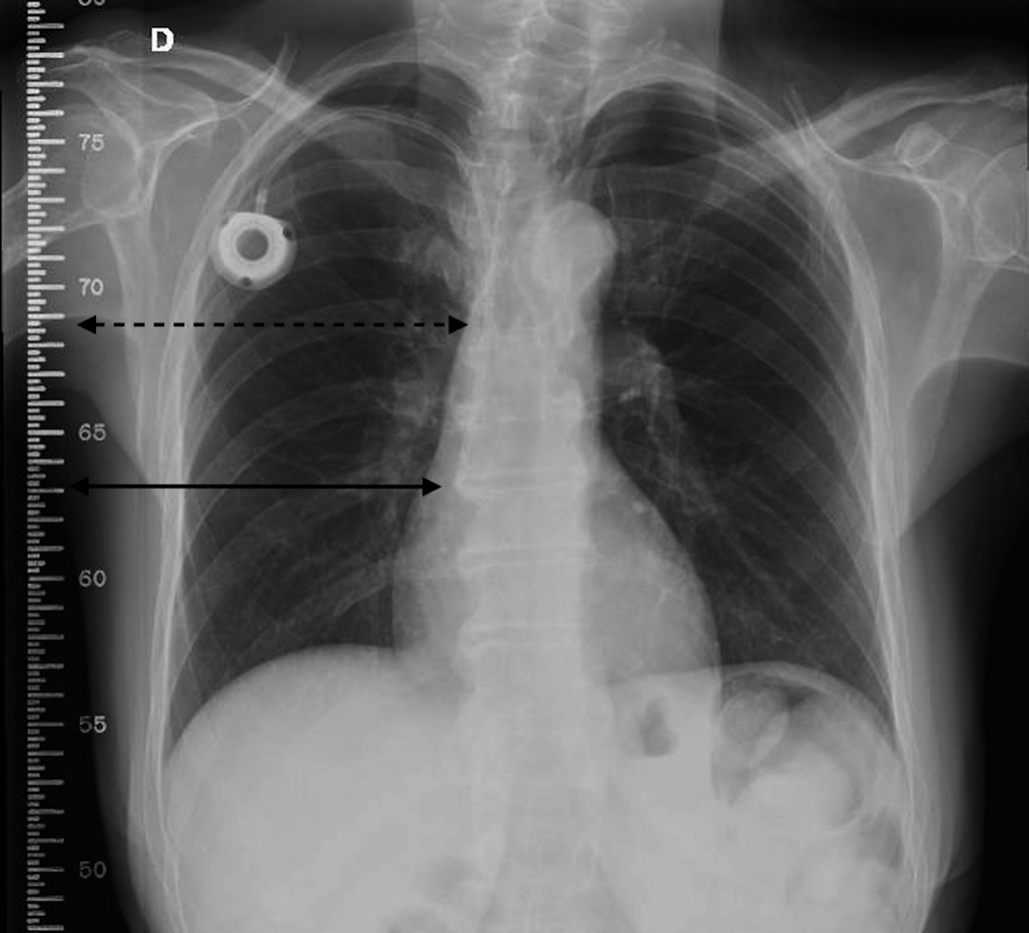
Postoperative standing X-ray image of the measurement of the catheter tip distance from the carina

The catheter tip in the upright position was assessed using routine postoperative chest radiography for posteroanterior incidence. A radiopaque ruler was included in the imaging field to measure the distance from the catheter tip to the carina in cm (Figure 2).

Plain arrow: catheter tip; Dashed arrow: carina.

### Data analysis and interobserver validation

Two trained observers, who were part of the procedural team, were assigned to measure the distance between the catheter tip and the carina in both the intraoperative (supine) and postoperative (upright) positions.

Catheter tip displacement was measured in mm. Negative values indicated displacement toward the carina (shortening), whereas positive values indicated displacement away from the carina (lengthening). For a comparative analysis, the mean tip displacement was calculated for both the mammary hypertrophy and Control Groups.

Observer 2's measurements were used to validate those of Observer 1 by calculating the intraclass correlation coefficient (ICC). The measurements obtained by Observer 1 were used as references for all subsequent statistical analyses. Prior to data collection, both observers completed a standardized training protocol to minimize inter-observer variability and ensure consistency in measurement techniques.

### Statistical analysis

Normal distribution of the data was confirmed using the Shapiro-Wilk test. Demographic and clinical characteristics are presented as relative frequencies and mean values with their respective standard deviations.

Qualitative characteristics related to catheter tip displacement were evaluated using Student's t-test, while quantitative characteristics were assessed in terms of TIVAD tip displacement using Pearson's correlation.

All statistical analyses were conducted using the SPSS software, version 25.0 (IBM Corp., Armonk, NY, USA).

Significance level was set at p<0.05.

## RESULTS

**Figure 3:**
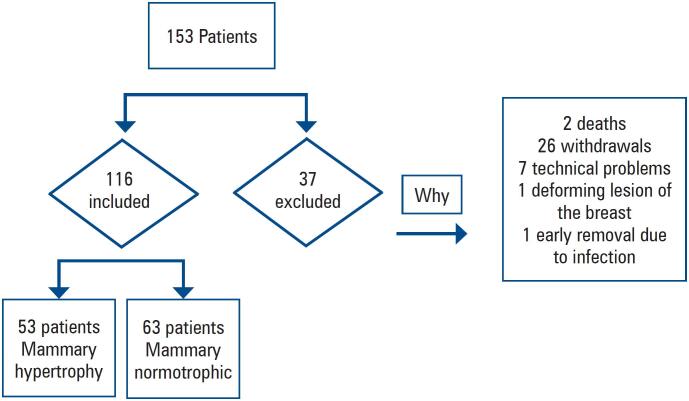
Patient enrolment flowchart

A total of 116 patients were included in the study. Patient enrolment is detailed in figure 3.

The average patient profile comprised individuals who were approximately 57 years old, overweight, and diagnosed with gastrointestinal neoplasms. The demographic and clinical characteristics of the participants are presented in table 1.

**Table 1:** Demographic and characteristics of patients

Variable		number and average	(%) / SD
Age (years)		57.52	±15.48
Weight (kg)		63.80	±17.87
Height (cm)		157.56	±7.16
BMI (kg/m^2^)		25.59	±6.5
Sacchini Index (mm)		107.17	±30.13
Neoplasia			
	Gastrointestinal	67	57.76
	Hematologic	18	15.52
	Breast	16	13.79
	Genitourinary	8	6.90
	Lung	4	3.45
	Musculoskeletal	3	2.59

SD: standard deviation; BMI: body mass index.

The ICC for catheter tip displacement measurements between Observer 1 and Observer 2 was excellent (*r*=0.773)^(12)^ indicating strong inter-observer agreement. The detailed values are listed in table 2.

**Table 2:** Inter-observer concordance for the catheter tip displacement

Variable	N	Average (mm)	SD	ICC	(95% CI)		Repeatability
					Lower	Higher	
Carina distance – catheter tip intraoperative (mm) OBS 1	116	47.1	14.5	0.853	0.795	0.896	5.71
Carina distance – catheter tip intraoperative (mm) OBS 2	116	46.8	15.2				
Carina distance – catheter tip orthostasis (mm) OBS 1	116	48.4	20.6	0.926	0.885	0.952	5.13
Carina distance – catheter tip orthostasis (mm) OBS 2	116	45.9	19.1				
Variation OBS 1	116	-1.32	15.36	0.712	0.607	0.792	7.73
Variation OBS 2	116	0.93	13.73				

ICC: intraclass correlation coefficient; SD: standard deviation; 95%CI: 95% confidence interval; OBS: observer.

Regarding catheter tip displacement (Table 3), statistically significant associations were observed with a history of non-deforming thoracic wall surgery (p=0.002) and the presence of a PICC during TIVAD implantation (p=0.042). Mammary hypertrophy was not significantly associated with catheter tip displacement during follow-up (p=0.182).

**Table 3:** Patient characteristics and correlation with tip displacement

Qualitative Characteristics		N	Average tip displacement (mm)	Standard Deviation	Median (mm)	Minimum (mm)		Maximum (mm)	p value
Previous surgery to the thoracic wall									0.002*
	No	112	-0.52	14.79	1.5	-38		31	
	Yes	4	-23.88	15.83	-24.75	-40		-6	
Presence of a PICC during procedure									0.042*
	No	85	-2.89	16.11	-5	-40		29	
	Yes	31	2.97	12.33	3	-18,5		31	
Previous catheter in the implanted vessel									0.357*
	No	103	-0.85	15.29	0	-40		31	
	Yes	13	-5.04	16.05	-1	-36		13.5	
Previous thrombosis on the implanted side									0.167*
	No	115	-1.14	15.30	0	-40		31	
	Yes	1	-22.50	0	-22.5	-22.5		-22.5	
Chest mass that causes deformity									0.559*
	No	110	-1.13	14.86	0	-38		31	
	Yes	6	-4.92	24.37	-1	-40		20	
Port implant side									0.923*
	Left	8	-0.81	16.27	2	-30		20	
	Right	108	-1.36	15.37	0	-40		31	
Group									0.182*
	Mammary hypertrophy	53	-3.41	16.16	-5	-40		27	
	Mammary normotrophic	63	0.43	14.55	2	-36		31	
Quantitative characteristics		Correlation					p-value		
	BMI	– 0.026					0.779#		
	Age (Years)	0.121					0.195#		
	Sacchini Index	-0.048					0.612#		

* Student's *t*-test;

# Pearson's correlation.

BMI: body mass index.

Mammary hypertrophy, assessed using the Sacchini index, did not significantly correlate with catheter tip displacement (p=0.612).

## DISCUSSION

TIVADs are widely used in patients who require long-term intravenous therapy. When properly implanted and managed, TIVADs are reliable and convenient tools for the prolonged administration of oncological treatments.^(2)^ Although techniques for TIVAD placement may vary depending on institutional protocols and patient-specific factors, the distal catheter tip should ideally be positioned at the cavoatrial junction within the superior vena cava.^(5)^ The reservoir should be anchored to a stable anatomical surface to ensure ease of access and facilitate long-term management,^(5)^ with the anterior thoracic wall, particularly in the infraclavicular deltopectoral groove, being the preferred site.^(1,13)^

The functionality of fully implantable catheters, particularly their ability to deliver medications and aspirate blood effortlessly, depends on proper placement of the reservoir, catheter tip, and subcutaneous tunnel.^(1,14)^ Despite their clinical utility, TIVADs are associated with complications that can seriously affect both the prognosis and quality of life in patients with cancer.^(1,2,15)^ Catheter thrombosis and infection are the two main complications associated with these devices, with the migration of the catheter tip acting as a significant risk factor for thrombosis and device malfunction.^(15-17)^

According to Vesely et al., in the supine position, mediastinal structures, including the central veins, are compressed by the abdominal contents.^(6)^ In the upright position, the abdominal contents shift caudally, leading to elongation of the central veins and expansion of the right atrium. Given that the proximal catheter segment remains fixed at the subcutaneous reservoir, this anatomical stretch may alter the position of the distal tip relative to the superior vena cava and the right atrium.^(6,7)^

An additional anatomical factor influencing the final position of the catheter tip is the tunnelling and implantation of the subcutaneous reservoir on the anterior thoracic wall. In the upright position, gravitational force causes caudal displacement of the thoracic wall under the influence of gravity.^(7)^ As the proximal segment is fixed to, this caudal shift can result in a cranial pull on the intravascular catheter tip, displacing it away from the cavoatrial junction.^(6-8)^

Contrary to the initial assumptions regarding the influence of breast volume, our analysis demonstrated that previous non-deforming breast surgery and the presence of a PICC at the time of TIVAD implantation were significantly associated with postoperative catheter tip displacement. Prior breast surgery was associated with an average caudal displacement of 23.88 mm, which was potentially related to anatomical alterations in the breast structure (modifying the volume-to-weight ratio) or fibrotic retraction of the thoracic wall. However, this finding should be interpreted with caution, as only four patients in the sample had a history of thoracic surgery.^(6-8)^

In contrast, the presence of a PICC at the time of TIVAD placement was associated with a mean cranial displacement of 2.97 mm. This may reflect mechanical interference within the vascular lumen or manipulation during the procedure, both of which can contribute to catheter tip migration.^(18)^

Mammary hypertrophy, as assessed using the Sacchini index, was not significantly associated with postoperative catheter tip displacement. This observation suggests that breast size alone may not be a reliable predictor of catheter movement, potentially due to confounding variables such as breast density, which could affect the gravitational pull on the thoracic wall, independent of volume.

Although our study provided valuable insights into catheter tip displacement after TIVAD implantation, several limitations must be acknowledged. First, it was conducted within a specific patient population and clinical setting, and these findings may not be directly generalizable to other healthcare environments or patient demographics. Second, although the sample size was sufficient for primary comparisons, it may have limited the ability to detect subtle associations. Furthermore, the impact of unmeasured confounding variables and the potential for interobserver variability in distance measurements, despite prior standardization, must be considered. Lastly, although our results revealed statistically significant associations, the clinical relevance and broader implications of catheter tip displacement require further investigation and validation in prospective studies.

Despite these limitations, this study provided valuable insights into a previously unexplored issue in clinical practice. Although we did not observe a higher incidence of catheter tip displacement in patients with mammary hypertrophy, our findings provide a foundation for future research and may serve as a practical reference for guiding future TIVAD implantation strategies. By identifying prior non-deforming thoracic factors associated with catheter tip displacement, this study enhances our understanding of potential risk factors and allows for more informed decision-making regarding catheter tip displacement in clinical settings.

## CONCLUSION

Mammary hypertrophy, as assessed using the Sacchini index, was not significantly correlated with the tip displacement of totally implantable venous access devices.

## Data Availability

The underlying content is contained within the manuscript.
